# Risk factors for violence among female forensic inpatients with schizophrenia

**DOI:** 10.3389/fpsyt.2023.1203824

**Published:** 2023-06-30

**Authors:** Viviane Wolf, Juliane Mayer, Ivonne Steiner, Irina Franke, Verena Klein, Judith Streb, Manuela Dudeck

**Affiliations:** ^1^Department of Psychiatry and Psychotherapy, Medical Faculty, LVR-Clinic Duesseldorf, Heinrich Heine University Duesseldorf, Duesseldorf, Germany; ^2^Department of Forensic Psychiatry and Psychotherapy, kbo-Isar-Amper-Clinic Taufkirchen (Vils), Taufkirchen (Vils), Germany; ^3^Psychiatric Services of Grisons, Chur, Switzerland; ^4^Department of Forensic Psychiatry and Psychotherapy, Ulm University, Guenzburg, Germany

**Keywords:** schizophrenia, violence, psychosis, violence risk assessment, female offenders

## Abstract

**Introduction:**

Schizophrenia is associated with a heightened risk of violent behavior. However, conclusions on the nature of this relationship remain inconclusive. Equally, the empirical evidence on female patients with schizophrenia spectrum disorders (SSD) is strongly underrepresented.

**Methods:**

For this purpose, the first aim of the present retrospective follow-up study was to determine the risk factors of violence in a sample of 99 female SSD patients discharged from forensic psychiatric treatment between 2001 and 2017, using three different measures of violence at varying time points (i.e., violent index offense, inpatient violence, and violent recidivism). Potential risk factors were retrieved from the relevant literature on SSD as well as two violence risk assessment instruments (i.e., HCR-20 V3, FAM). Further, we aimed to assess the predictive validity of the HCR-20 V3 in terms of violent recidivism and evaluate the incremental validity of the FAM as a supplementary gender-responsive assessment.

**Results:**

The given results indicate strong heterogeneity between the assessed violence groups in terms of risk factors. Particularly, violence during the index offense was related to psychotic symptoms while inpatient violence was associated with affective and behavioral instability as well as violent ideation/intent, psychotic symptoms, and non-responsiveness to treatment. Lastly, violent recidivism was related to non-compliance, cognitive instability, lack of insight, childhood antisocial behavior, and poverty. Further, the application of the HCR-20 V3 resulted in moderate predictive accuracy (AUC = 0.695), while the supplementary assessment of the FAM did not add any incremental validity.

**Discussion:**

This article provides important insights into the risk factors of violence among female SSD patients while highlighting the importance of differentiating between various forms of violence. Equally, it substitutes the existing evidence on violence risk assessment in female offenders with SSD.

## 1. Introduction

In the public eye, people with severe mental disorders are frequently associated with violent behavior. Particularly, people suffering from psychotic disorders are believed to engage in violent offenses at disproportionally high rates, thereby being majorly affected by stigmatization (1). Given a 10% lifetime prevalence of violence among patients with schizophrenia spectrum disorders (SSD), this belief is clearly overestimating the occurrence of violence in SSD (2). However, compared to the general population, people diagnosed with SSD have an increased probability (1–7 times) of acting violently throughout their life. For women, the odds even rise to 4–29 times (3). Alarmingly, these observations especially count for severe violence. Among homicide offenders, the percentage of offenders with SSD amounts to 5 to 20% (4), while the lifetime prevalence of schizophrenia in the general population roughly amounts to 0.7% (5). Consequently, the relevance of identifying the underlying risk factors of violence in SSD is clearly recognizable and particularly emphasized for female offending populations.

Reviewing the current state of literature, the most established risk factors of violence in people with SSD concern static risk domains. Specifically, factors related to criminal histories (6) such as previous violence (7–9), childhood misconduct (10, 11), and prior convictions were repeatedly found to be relevant predictors. Furthermore, substance (6, 8, 10–15) and alcohol abuse (6, 7) as well as comorbid psychiatric diagnoses were found to be related to higher rates of violence in patients with psychosis (16). Especially, being diagnosed with psychopathy (9, 15), personality disorders (17), or other personality pathologies (18) is associated with a heightened tendency to violent behavior and violent recidivism (19) in psychosis patients. Further, some studies suggest the association between violence and psychosis is negatively impacted by poverty and social disadvantages (10, 20, 21). More recent lines of research highlight the effect of dynamic risk factors, such as psychopathological and clinical factors. Particularly, positive psychotic symptoms including hallucinations and delusions were found to increase the violence risk (11, 16, 18, 22). On the contrary, negative symptoms such as social withdrawal or blunted affect (10, 11) were found to be protective factors of violence. Likewise, promising findings have been linking violence to clinical variables including non-adherence to treatment (6, 23, 24), poor insight (9, 15, 25, 26) and impulsivity (9, 15). In their review, Steinert (27) concluded that clinical and psychopathological variables are more predictive for inpatient violence, while static risk factors are more applicable to community violence. With regard to the existing literature on violence in female schizophrenia patients, some differences appear in comparison to the male gender. While previous violence, prior convictions, and victimization were found to elevate the violence risk for both genders, violence in female schizophrenia patients was particularly associated with the diagnosis of a Cluster B personality disorder as well as high levels of unmet needs (28).

However, while scientific literature has repeatedly supported a moderate but significant link between schizophrenia and violence, little consensus exists on the nature of this relationship (3, 18). Due to the strong methodological variation in the existing literature, reliable conclusions on the risk factors of violence in SSD patients remain limited. To name a few, methodological differences concern the implementation of violence and schizophrenia, the study design, and the impact of confounding factors (1, 18). Especially regarding the conceptualization of violence, fundamental differences appear. For instance, some studies define violence as threats or verbal aggression, while others only address physical violence. Equally, several studies measure violence on a dichotomous scale, whereas others assess different levels of severity. Further, studies have been using various designs, including retrospective, prospective, and cross-sectional designs. Also, some studies have controlled for general risk factors of violence, such as substance use or psychopathy, while others did not account for any confounding factors (18).

Additionally, the existing literature comes with some relevant shortcomings, which further strengthen the relevance of studying violence in female offenders with SSD. Primarily, studies on the risk factors of violence in SSD mainly include male samples, while studies on women are nearly non-existent (28). Given that the male gender itself represents a major risk factor for violence and the proportion of women engaging in violent acts is clearly outnumbered, the generalizability of determined risk factors to female offenders needs to be questioned (29). Similarly, research has repeatedly shown that the risk of violence in people with psychosis is elevated for females (28). Further, the given literature on the risk factors of violence in psychiatric patients predominantly addresses community samples, while only a minor proportion focuses on forensic or inpatient samples. However, community samples as well as forensic/civil inpatient samples differ fundamentally in terms of observation periods, the availability of drugs/alcohol, pharmacological treatment, the presence of acute symptoms, and comorbidities with antisocial personality disorder (27). Equally, various countries have reported rising admission rates for female offenders in forensic psychiatric care as well as a strong increase in violent offenses (29). It therefore appears essential to determine the predictors of violence in female forensic inpatients with SSD. Each lacking empirical evidence, it is particularly important to assess violence at the different stages of forensic treatment, including violent offending leading to admission, inpatient violence, and violent re-offending after being discharged from forensic psychiatric care.

Within forensic psychiatric treatment, the assessment of patients’ violence risk constitutes an essential step in determining the adequate focus and duration of treatment (29). To standardize this process, create more transparency, and enhance predictive accuracy, it has become common practice to use violence risk assessment instruments. Following scientific recommendations, structured professional judgment tools are currently considered best practice. This approach centrally entails combining actuarial and clinical judgment in order to determine a patient’s violence risk and accordingly select the appropriate risk management plan. Briefly, actuarial judgment uses statistical models to identify risk factors that enhance the likelihood of future offending/violence, while clinical judgment relies on professional expertise to determine an individual risk of violence/reoffending (30). As the prevailing instrument, the Historical and Clinical Risk Management, Version 3 (HCR-20 V3) (31) has been validated in various populations. Divided into three distinct sections, the instrument assesses historical variables, clinical variables, and risk management variables. While the historical domain addresses static risk factors (i.e., unchanging and stable over time), the clinical and risk management domains assess dynamic risk factors (i.e., changeable and possibly fluctuating over time/with treatment). Textually, the HCR-20 V3 covers the central risk factors of violence in mentally disordered offenders (e.g., previous violence, substance use, psychopathy/personality disorders, active symptoms, insight, treatment responsiveness, and impulsivity). Reflecting recent criticism of current risk assessment instruments, the included risk factors are derived from predominantly male samples. In response, proponents of the feminist perspective have highlighted the need to consider risk factors specific to female offenders (32, 33). For the gender-responsive prediction of violent reoffending, the female additional manual (FAM) (34) was developed as a supplement to the HCR-20 V3. However, little research has been conducted to verify its predictive accuracy, while primary studies could merely support its clinical relevance (29). Further, both instruments were created for risk assessment in mentally disordered patients in general without differentiating specific diagnoses. Among other psychiatric disorders, the HCR-20 V3 is assumed to be applicable to schizophrenia patients, but this notion is still lacking evidence (35, 36). Therefore, more extensive and sophisticated research is needed to verify the applicability of existing violence risk assessment instruments, both male-based and gender-responsive, in female schizophrenia patients. At the same time, it is needed to investigate the unique correlates of violence presented by this population.

The aims of the present retrospective follow-up study were: (1) to determine the risk factors of violence in female forensic inpatients diagnosed with schizophrenia spectrum disorders, (2) to assess the predictive validity of the HCR-20 V3 as the prevailing male-based violence risk assessment tool in terms of violent recidivism, and (3) to evaluate the incremental validity of the FAM as a gender-responsive supplement to the HCR-20 V3. Potential risk factors were retrieved from the available literature on SSD as well as the two risk assessment instruments. To account for violence throughout the different stages of forensic treatment (i.e., prior to admission, during treatment, and after discharge), three independent variables were implemented covering these time points (i.e., violent/non-violent index offense, inpatient violence/no inpatient violence, and violent/no violent recidivism). Accordingly, risk factors for each measure of violence were assessed separately.

## 2. Materials and methods

### 2.1. Participants

The study sample consisted of 99 female forensic psychiatric inpatients diagnosed with schizophrenia spectrum disorders. According to ICD-10 criteria, we included patients diagnosed with schizophrenia, delusional disorder, acute polymorphic psychotic disorder, and schizoaffective disorder. The included sample was a complete survey of all female forensic inpatients with SSD discharged from forensic psychiatric care in Bavaria, Germany between 2001 and 2017. Eight patients had to be excluded beforehand due to death or emigration. To be included in the study, patients had to meet the following criteria: (1) minimum age of 18 and (2) a final conviction for admission to a forensic psychiatric facility. All the patients were ordered by court into forensic psychiatric treatment, either according to section 63 or section 64 of the German Penal Code. Treatment according to section 63 is unlimited in duration but annually reviewed. It is applied if a major mental illness is assumed to be centrally linked to the initial offense. It requires a diminished criminal responsibility and a prevailing risk of reoffending. Treatment according to section 64 is time-limited and applied when a substance use disorder is considered decisive for the initial offense. It requires a positive treatment prognosis and a prevailing risk of reoffending. The duration of treatment varies based on additional prison sentences but generally amounts to two years. If the requirements of successful treatment completion are no longer fulfilled, Section 64 patients may return to prison.

### 2.2. Materials

#### 2.2.1. Historical clinical risk management-20, version 3

The HCR-20 V3 is a standardized risk assessment tool used to predict future violence in mentally disordered offenders and accordingly suggest adequate risk management strategies. The instrument includes 20 risk factors, sectioned into three textual domains. The “historical” (H) domain includes 10 items, referring to problems in the past (i.e., violence, antisocial behavior, relationships, employment, substance use, major mental disorders, personality disorders, traumatic experiences, violent attitudes, and treatment/supervision response). The “clinical” (C) domain includes five items relating to problems in the last six months (i.e., insight, violent ideation/intent, symptoms of a major mental disorder, instability, and treatment/supervision response). Lastly, the “risk management” (R) domain includes five items concerning expected problems in the upcoming six months (i.e., professional services/plans, living situation, personal support, treatment/supervision response, stress/coping). For the German Version of the HCR-20 V3, Dahle (37) found a moderate predictive accuracy for general recidivism (*r* = 0.24) and a good predictive ability for violent recidivism (*r* = 0.36). Von Franque (38) determined a median interrater reliability of 0.65 (range: 0.41–0.76), which is considered good to excellent.

#### 2.2.2. Female additional manual

The FAM is a user guide designed to supplement the HCR-20 V3 with gender-responsive risk factors and additional guidelines adjusted to the violence risk of female offenders with mental disorders. The instrument consists of eight additional risk factors and two supplementary guidelines. The risk factors include four historical factors (i.e., prostitution, parenting difficulties, pregnancy at a young age and suicidality/self-harm), two clinical factors (i.e., covert/manipulative behavior and low self-esteem), and two risk management factors (i.e., problematic childcare responsibility and problematic intimate relationship). A good interrater reliability (ICC = 0.63–0.97) was found for the individual items of the FAM and the additional guidelines to the HCR-20 V3 (34).

### 2.3. Procedure

As part of a larger research project on the feasibility of common risk assessment tools in mentally disordered female offenders, the given study focused on the subgroup of female forensic inpatients diagnosed with schizophrenia spectrum disorders. For the purposes of identifying the relevant risk factors for this subgroup, a wide range of factors was assessed in the given study. Primarily, common risk factors of violence in mentally disordered offenders were assessed. As the prevailing instrument that covers these risk factors, the items of the HCR-20 V3 were incorporated. Further, to account for gender-responsive risk factors of violence in mentally disordered offenders, the items of the FAM were included. In the next step, the existing literature was reviewed to select risk factors of violence specific to patients with schizophrenia spectrum disorders. While a significant proportion was already covered by the instruments, we additionally included sociodemographic information (e.g., age, educational achievement, poverty), criminal history data (e.g., number of prior convictions) as well as clinical variables including psychiatric diagnoses (e.g., alcohol/substance use disorders, depression, personality disorders) and psychopathological symptoms (i.e., positive psychotic symptoms, alcohol/substance intoxication). Additionally, treatment-specific measures were included (i.e., treatment duration, time at risk). Diagnoses were predefined at discharge according to the diagnostic criteria of the ICD-10. For the remaining items, definitions were retrieved from the relevant literature. The violence risk assessment instruments were rated specifically for this study. In preparation for the coding of the instruments, each of the five raters was professionally trained. To evaluate the interrater agreement on the risk assessment instruments, interrater reliability testing was carried out. For the HCR-20 V3 moderate results (ICC = 0.606, 95%-CI = 0.345; 0.845) were gathered, while good results were found for the FAM (ICC = 0.818, 95%-CI = 0.638; 0.938). For the rating of the items, which was conducted retrospectively, file information was retrieved from patient records including official court documents. To account for the variation in quality and completeness of the files, only those patient files were included that covered the necessary information to accurately rate the items. To be included in the study, at least the court decision/the forensic psychiatric assessment at admission and a report on the therapeutic process at discharge had to be available. The independent variables (i.e., violent index offense, inpatient violence and violent recidivism) were coded on a binary scale (yes/no). Violence was assessed according to the definition of violence provided in the HCR-20 V3, being the “actual, attempted, or threatened infliction of bodily harm of another person” (31). The index offense was defined as the offense decisive of admission to forensic psychiatric treatment. Correspondingly, any index offense, where violence was reported, was coded as violent index offense. Any documented violence during forensic treatment was coded as inpatient violence and any violent reconviction was rated as violent recidivism. For the assessment of violent reoffending, extracts from the Federal Central Criminal Register were demanded in September 2020 and February 2021. Data was collected between 2019 and 2021. Study approval was obtained by the Ethics Committee of the Bavarian Medical Association (approval no. 2019–167).

### 2.4. Data analysis

For all statistical analyses, IBM SPSS Statistics Version 29 was used. Missing data was very limited (ranging from 0 to 1% across the included variables) and randomly distributed (MCAR). Therefore, no missing data was imputed and any subjects with missing values on any of the assessed variables were excluded from the analysis. First, for each independent variable (i.e., violent index offense, inpatient violence, and violent recidivism) comparisons were conducted between the violent and non-violent subjects to determine differences in possible risk factors. For binary predictors, chi-square tests of independence were conducted. As a measure of effect size, Cramer’s V was used. According to Cohen (39), the following interpretations were applied: below 0.10 was considered a small effect, between 0.10 and 0.30 was considered medium, and above 0.30 was regarded as a large effect. For continuous predictors, Mann–Whitney-U-Tests were applied. The HCR-20 V3 risk factors were originally coded on a 3-point scale (risk factor is present/partially present/not present). To account for the small sample sizes of the violent groups and reduce low cell sizes, the HCR-20 V3 items were recoded into dichotomous variables (risk factor is present/not present). For the same reason, the determined risk factors were not analyzed in a multivariate model. Lastly, receiver operating characteristic (ROC) analysis was carried out to calculate the predictive accuracy of the HCR-20 V3 and the FAM with regard to violent reoffending. Following the guidelines by Rice and Harris (40), AUC values above 0.56 were considered a small effect, values above 0.64 a medium effect, and values above 0.71 a large effect.

## 3. Results

### 3.1. Sample characteristics

For the total study sample of female forensic inpatients with schizophrenia spectrum disorders (*N* = 99), the mean age at admission was 39.05 years (range: 18–67 years). The mean duration of inpatient treatment was 54.91 months (range: 5–152 months) and the mean follow-up period was 10.13 years (range: 3.05–19.61 years). 80.8% of the patients were admitted according to section 63 of the German Penal Code, while 19.2% were admitted according to section 64. Regarding comorbid diagnoses at the time of the discharge from forensic care, 42.9% were provided with the diagnosis of a substance use disorder (ICD-10, F1), with 21.4% being diagnosed with an alcohol use disorder. Further, 1% were diagnosed with an affective disorder (ICD-10, F3) and 5.1% were given the diagnosis of an eating disorder (ICD-10, F5). Lastly, 10.1% were diagnosed with a personality disorder (ICD-10, F6), i.e., 8.1% were given an emotionally unstable personality disorder and 2% a mixed personality disorder. Regarding the initial offenses, 80.8% of the sample had committed a violent offense causal to admission to the forensic facility. Among the violent offenders, 32.3% of the patients were convicted of bodily harm, 23.2% of homicide, 13.1% of arson, 7.1% of robbery, and 5.1% of unlawful detention/threat/coercion. Among the non-violent offenders, 14.1% were convicted of property offenses, 2% of drug-related offenses, and 3% of other, non-violent offenses. Further, 22.2% of the sample showed inpatient violence during forensic psychiatric treatment, while 21.2% reoffended after discharge from forensic psychiatric care and 10.1% re-offended with a violent offense.

### 3.2. Risk factors of violence

#### 3.2.1. Violent index offense

As displayed in Table 1, violence during the index offense was significantly associated with several static variables including age, the number of prior convictions, antisocial behavior during adolescence (HCR-20 H1b), lower educational achievement (i.e., school leaving certificate below grade 10), and substance use (HCR-20 H5). Each significant factor showed a medium effect size. Further, a significant association was found between violence during the index offense and the dynamic risk factor positive psychotic symptoms during index offense. Other factors related to sociodemographic information, clinical variables as well as gender-responsive risk factors (FAM) were not significantly related to violence during the index offense.

**Table 1:** Significant differences between female patients convicted of a violent index offense and those convicted of a non-violent index offense.

	Violent *n* (%)/*M* (SD)	Non-violent *n* (%)/*M* (SD)	*X*^2^/*U*	Cramer’s V
Age (during index offense)	39.71 (12.01)	30.53 (10.81)	415.500**	–
Number of prior convictions	1.93 (4.16)	4.47 (3.19)	317.000***	–
Antisocial behavior as an adolescent (HCR-20 H1b)	22 (27.8%)	12 (63.2%)	8.428**	0.293
Substance use (HCR-20 H5)	36 (45%)	14 (73.7%)	5.054*	0.226
Lower educational achievement	40 (50.6%)	15 (78.9%)	4.986*	0.226
Positive psychotic symptoms (during index offense)	67 (83.8%)	10 (52.6%)	8.602*	0.295

*p < 0.05, **p < 0.01, ***p < 0.001.

#### 3.2.2. Inpatient violence

As presented in Table 2, inpatient violence was significantly related to various clinical risk factors (past 6 months) including violent ideation or intent (HCR-20 C2), affective instability (HCR-20 C4a), behavioral instability (HCR-20 C4b), and non-responsiveness to treatment or supervision (HCR-20 C5b). Equally, positive psychotic symptoms (past 12 months) significantly differentiated between patients that showed inpatient violence and those that did not. While affective instability and positive psychotic symptoms had a medium effect size, behavioral instability, non-responsiveness to treatment, and violent ideation or intent were accompanied by a high effect size. Other risk factors including sociodemographic information (e.g., age), criminal history, treatment-related factors, clinical variables as well as gender-responsive risk factors (FAM) did not differentiate significantly between the groups.

**Table 2:** Significant differences between female patients who showed inpatient violence and those who showed no inpatient violence.

	Violent *n* (%)	Non-violent *n* (%)	*X*^2^	Cramer’s V
Violent ideation or intent (HCR-20 C2)	3 (13.6%)	0 (0%)	10.828*	0.331
Affective instability (HCR-20 C4a)	12 (54.5%)	21 (27.3%)	5.727*	0.241
Behavioral instability (HCR-20 C4b)	14 (63.6%)	19 (24.7%)	11.688***	0.344
Non-responsiveness to treatment (HCR-20 C5b)	17 (77.3%)	29 (37.7%)	10.793**	0.330
Positive psychotic symptoms (past 12 months)	10 (45.5%)	15 (19.5%)	6.116*	0.249

*p < 0.05, **p < 0.01, ***p < 0.001.

#### 3.2.3. Violent recidivism

As shown in Table 3, violent recidivism was significantly associated with the historical/static risk factors number of prior convictions, antisocial behavior during childhood (HCR-20 H2a) and poverty. Further, clinical risk factors including lack of insight into treatment needs (HCR-20 C1c) and cognitive instability (HCR-20 C4c) showed significant associations with violent recidivism. Lastly, the risk management factor non-compliance with treatment or supervision (HCR-20 R4a) significantly differentiated between violent and non-violent (re-)offenders. Each of the determined risk factors was accompanied by a medium effect size. Other factors including sociodemographic information (e.g., age), treatment-related factors (i.e., treatment duration, time at risk), further clinical variables as well as gender-responsive factors (FAM) were not significantly related to violent reoffending.

**Table 3:** Significant differences between female patients with violent recidivism and those without violent recidivism.

	Violent *n* (%)/*M* (SD)	Non-violent *n* (%)/*M* (SD)	*X*^2^/*U*	Cramer’s V
Number of prior convictions	4.40 (4.58)	2.19 (4.02)	276.500*	–
Antisocial behavior as a child (HCR-20 H2a)	3 (30%)	6 (6.8%)	5.786*	0.243
Lack of insight into need for treatment (HCR-20 C1c)	8 (80%)	38 (42.7%)	5.029*	0.225
Cognitive instability (HCR-20 C4c)	8 (80%)	39 (43.8%)	4.719*	0.218
Non-compliance with supervision (HCR-20 R4a)	10 (100%)	48 (53.9%)	7.863**	0.282
Poverty	10 (100%)	49 (55.1%)	7.541**	0.276

*p < 0.05, **p < 0.01, ***p < 0.001.

### 3.3. Predictive validity of HCR-20 V3 and FAM

Receiver operating characteristic (ROC) analysis was carried out to determine the predictive accuracy of the HCR-20 V3 and the FAM. As presented in Table 4, the HCR-20 V3 significantly predicted violent recidivism. The area under the curve (AUC) indicates a moderate effect (AUC 0.64–0.71). When combining the HCR-20 V3 and the FAM, the prediction of violent recidivism no longer remained statistically significant and the AUC value slightly decreased, indicating that the addition of the FAM lowered predictive accuracy. When applying the FAM without additional assessment of the HCR-20 V3, the AUC value further decreased. The FAM was not able to significantly discriminate between the violent and non-violent group.

**Table 4:** AUC analysis used to assess the predictive accuracy of the HCR-20 V3 and the FAM.

	AUC	CI 95%	Standard error	*p*
HCR-20 V3	0.695	(0.581, 0.809)	0.058	0.044
FAM	0.572	(0.387, 0.758)	0.095	0.454
HCR-20 V3 and FAM combined	0.682	(0.546, 0.818)	0.069	0.060

## 4. Discussion

The aim of the given study was to provide evidence on the link between schizophrenia and violence in the population of female forensic inpatients. Primarily, the predictors outlined in this article are showing a strong focus on dynamic risk factors. Particularly, patients’ psychopathological status and their treatment response were proven of central relevance. With regard to static risk factors, the given study has produced conflicting findings, specifically concerning the role of criminal history, previous violence and substance abuse. Further, this article is supporting the applicability of the HCR-20 V3 for convicted female SSD patients, which implies that the population of forensic inpatients with psychosis presents comparable risk factors to the population of mentally ill offenders in general. Lastly, the results are highlighting the strong variation among the risk factors of violence, depending on the measure that is used (i.e., violent index offense, inpatient violence, or violent recidivism), which supports the heterogeneity within this population outlined in previous research (18).

Concerning violence during the index offense, which was the case for 80.8% of the sample, the only risk factor that could be identified in the given study concerned psychotic symptoms during the initial offense. However, some protective factors of violence during the index offense were found. These included antisocial behavior during adolescence, lower educational achievement, and a history of substance use. Primarily, these findings correspond with previous studies that found positive psychotic symptoms to enhance the violence risk (11, 22, 24). However, with regard to the protective effect of the remaining factors, the current findings are contradicting earlier findings which highlight the negative effect of substance use, lower educational achievement (8) and adolescence misconduct on violence in patients with schizophrenia spectrum disorders (41).

With regard to inpatient violence, which accounted for 22.2% of the sample, risk factors exclusively concerned dynamic clinical factors, addressing the patients’ psychopathological status of the past 6–12 months. Specifically, non-responsiveness to treatment, affective and behavioral instability, positive psychotic symptoms, and violent ideation or intent could significantly differentiate between violent and non-violent inpatients. These findings partially support previous findings. Primarily, they correspond with a review by Steinert (27) on the risk factors of inpatient violence, which emphasized the focus on clinical and psychopathological variables (i.e., psychotic symptoms and hostility). Further, they are in line with previous studies on the risk factors of inpatient violence in SSD patients which found positive symptoms, hostility (22), impulsivity (9, 22), and negative attitudes (original HCR-20 V3 equivalent to violent ideation or intent) (9) to be related to inpatient violence. However, the review by Steinert (27) also stressed that prior violence consistently predicted inpatient violence among three studies on psychiatric patients, which was not the case for the present study. This finding indicates that the importance of dynamic, clinical variables, which was evident for inpatient violence in psychiatric patients, is even further enhanced for the population of female forensic inpatients with SSD.

Lastly, violent recidivism, which concerned 10.1% of the sample, presents a rather mixed picture of risk factors. On the one hand, static risk factors including antisocial behavior during childhood, a higher number of prior convictions and experiences of poverty were associated with violent reoffending. This is consistent with prior literature, which found childhood conduct problems to enhance the probability of violence in patients with SSD (10, 11). Equally, previous studies have shown the negative effect of poverty, homelessness, and social deprivation (10, 20, 21) on violence and violent recidivism. On the other hand, dynamic risk factors including lack of insight into treatment needs, cognitive instability, and non-compliance with treatment/supervision were significantly associated with violent recidivism. These findings correspond with previous studies that support the negative effect of diminished insight (9, 15, 22, 25, 26), instability (9, 15), and non-compliance on violence (6, 23, 24). These findings also substantiate the feasibility of the HCR-20 V3 for female patients with SSD as the majority of the determined risk factors is covered by the instrument (i.e., antisocial behavior during childhood, non-compliance with treatment/supervision, lack of insight into need for treatment, and cognitive instability). Merely experiences of poverty are not included in the instrument.

In sum, these findings highlight the strong heterogeneity within the population of violent female SSD patients, which has been stressed by prior studies on male samples (18). While violence during the index offense was most closely related to acute psychotic symptoms and the absence of general risk factors of offending, inpatient violence seemed to occur in response to chronic, treatment-resistant positive psychotic symptoms. Violent reoffending, on the contrary, appeared to be accompanied by non-compliance and a stable pattern of antisocial behavior, starting in childhood, rather than psychosis-related risk factors. Accordingly, the influence of criminal history factors produced contrasting results depending on the assessed measure of violence. While criminal history factors (i.e., adolescence misconduct, number of prior convictions) were found to be protective factors for violence during the index offense, similar factors (i.e., childhood misconduct, number of prior convictions) were determined risk factors for violent reoffending. Given that the criminal histories differ in onset, this finding corresponds with a widely accepted developmental taxonomy by Moffitt (42), suggesting the existence of two distinct onset-based offending trajectories. As originally proposed, offenders that start expressing antisocial behavior during childhood are typified as presenting a stable and chronic offending pattern throughout their life, while adolescence onset offenders are expected to commit offenses less frequently and for a limited duration (42). Equally, these findings are consistent with Hodgins (43) who proposed two distinct offending typologies among offenders with major mental disorders. While those labeled as early starters closely resemble the description of Moffitt’s childhood onset offenders, late starters are suggested to offend in response to emerging symptoms of their mental disorder. For the early starters, offending is hypothesized to be unrelated to the mental disorder and instead driven by a stable antisocial behavior pattern, while offending in late starters is believed to be centrally liked to patients’ mental disorder. Accordingly, the continued violent offending observed in the given sample of female SSD patients may find its origin in the early offending onset, matching the description of Moffitt’s childhood onset offenders or Hodgins’ early starters. This conclusion aligns with prior literature on SSD, suggesting two separate patterns of violence. While one pattern of violence is believed to result from acute psychopathology, the other is proposed to follow antisocial behavior that onsets in childhood (44). Adding empirical support to this claim, Swanson (45) found that violence in SSD patients with a history of childhood antisocial behavior was statistically unrelated to symptoms of psychosis, while violence in SSD patients without childhood antisocial behavior was strongly associated with psychotic symptoms. Equally, these finding have promising implications on the violence risk directly related to psychotic symptoms. Particularly, if addressed by adequate treatment and risk management strategies during and after forensic treatment, the probability of violent reoffending related to psychotic symptoms appears to be strongly reduced. This goes in line with Franke (46), who found comparably low violent and non-violent reoffending rates in a sample of SSD patients in German forensic psychiatry, suggesting the effectiveness of forensic in-patient treatment and adequate aftercare is especially emphasized for patients with schizophrenia.

Notwithstanding the confirmative findings generated in the present study, considerable inconsistencies emerged in regard to the existing research on predominantly male samples. As mentioned earlier, the risk factors outlined in the given study mainly concern dynamic risk factors. Previous literature, however, has pinpointed several important static risk factors including previous violence (7–10), substance abuse (6, 8, 10–15), and victimization (10, 11, 28). In the present study, the measures of violence were not associated with any form of previous violence. Furthermore, substance abuse was found a protective factor of violence during index offenses. In the case of inpatient violence or violent recidivism, substance use showed no significant associations. Regarding experiences of victimization, no significant associations were found concerning any of the measures of violence.

Further, receiver operating characteristic (ROC) analysis showed moderate predictive accuracy of the HCR-20 V3, while the supplementary application of the FAM did not add incremental validity to the prediction of violent recidivism. This finding corresponds with prior literature (29, 47). Despite the demonstrated feasibility of the HCR-20 V3, a closer look at the results of the separate items may have some important implications for the given sample. Particularly, several risk factors that were found relevant for the original sample of the HCR-20 V3 did not predict violence in the given sample. Neither did the historical items contribute much to the prediction of violence (apart from antisocial behavior during childhood/adolescence), nor did the risk management factors (apart from non-compliance with treatment/supervision) explain much of the variance in violent recidivism. Therefore, it may be beneficial to specifically attend to the clinical factors of the HCR-20 V3 when predicting violence in female forensic inpatients with SSD.

Several limitations need to be considered. First, our data was record-based and retrospective in nature. Consequently, the data was not collected for the purpose of this study but was derived from pre-existing forensic documentation. Therefore, we were not able to confirm the accuracy of the information, and records differed in terms of content and completeness. Second, as we had to rely on external assessments, it was not possible to capture the patients’ perspective on the included measures. Future research may be advised to include a mixed methods design to address this shortcoming. Third, violent recidivism was assessed by officially registered offenses only. Using self-report data or other sources of information, more reliable inferences may have been possible. Despite these limitations, the given study comes with a major strength. Namely, the presented inferences were drawn from a comparably high sample size for this specific population. In fact, the included sample represents a complete survey of all female patients with SSD treated in a forensic psychiatric facility in Bavaria, Germany, for a total period of 17 years (2001–2017). Further, by retrieving the assessed risk factors from a standardized, validated risk assessment instrument, we addressed limitations of similar studies while improving the reliability of our conclusions.

## 5. Conclusion

The given study provides important evidence for the prediction of violence in female forensic inpatients with schizophrenia spectrum disorders. First, the heterogeneity within the population of violent SSD patients was highlighted, revealing distinct sets of risk factors for each violence group (i.e., violent index offense, inpatient violence, violent reoffending). While violence during the index offense was linked to positive psychotic symptoms, inpatient violence was related to a range of clinical factors including positive psychotic symptoms, violent ideation/intent, non-responsiveness to treatment as well as affective and behavioral instability. Lastly, violent reoffending was associated with a combination of static and dynamic risk factors including antisocial behavior during childhood, a higher number of prior convictions, experiences of poverty, lack of insight into treatment needs, non-compliance with treatment/supervision, and cognitive instability. Correspondingly, these findings suggest the existence of different patterns of violence in female SSD patients, one occurring due to acute psychotic symptoms and the other originating from early-onset antisocial behavior. Further, this study supports the applicability of the HCR-20 V3 for the given population, as ROC analysis showed a moderate predictive accuracy of the HCR-20 V3, while the additional assessment of the Female Additional Manual did not result in any improvement of the predictive accuracy.

## Data availability statement

The raw data supporting the conclusions of this article will be made available by the authors, without undue reservation.

## Ethics statement

The studies involving human participants were reviewed and approved by Ethikkommission der Bayerischen Landesärztekammer. Written informed consent for participation was not required for this study in accordance with the national legislation and the institutional requirements.

## Author contributions

MD, IF, and VK: conceptualization. VW and JS: methodology. VW: formal analysis. VW, JM, and IS: investigation and data curation. VW: writing—original draft preparation and writing—review and editing. MD and VK: funding acquisition. All authors have read and agreed to the published version of the manuscript.

## Funding

As part of a broad research project on risk assessment in female offenders, this research was funded by the Bavarian State Ministry of Families and Social Affairs (ZFBS), Office of Corrections, with a grant of EUR 420,300 (grant number ZBFS-X/1–10.700-5/3/9). This grant financed the work of VW, JM, and IS.

## Conflict of interest

The authors declare that the research was conducted in the absence of any commercial or financial relationships that could be construed as a potential conflict of interest.

## Publisher’s note

All claims expressed in this article are solely those of the authors and do not necessarily represent those of their affiliated organizations, or those of the publisher, the editors and the reviewers. Any product that may be evaluated in this article, or claim that may be made by its manufacturer, is not guaranteed or endorsed by the publisher.
